# Validation of an algorithm to identify children with biopsy-proven celiac disease from within health administrative data: An assessment of health services utilization patterns in Ontario, Canada

**DOI:** 10.1371/journal.pone.0180338

**Published:** 2017-06-29

**Authors:** Jason Chan, David R. Mack, Douglas G. Manuel, Nassim Mojaverian, Joseph de Nanassy, Eric I. Benchimol

**Affiliations:** 1 School of Epidemiology, Public Health and Preventive Medicine, University of Ottawa, Ottawa, Ontario, Canada; 2 Division of Gastroenterology, Hepatology and Nutrition, Children’s Hospital of Eastern Ontario, Ottawa, Ontario, Canada; 3 Department of Pediatrics, University of Ottawa, Ottawa, Ontario, Canada; 4 Institute for Clinical Evaluative Sciences, Ottawa, Ontario, Canada; 5 Ottawa Hospital Research Institute, Ottawa, Ontario, Canada; 6 Department of Pathology and Laboratory Medicine, University of Ottawa, Ottawa, Ontario, Canada; University of Illinois-Chicago, UNITED STATES

## Abstract

**Importance:**

Celiac disease (CD) is a common pediatric illness, and awareness of gluten-related disorders including CD is growing. Health administrative data represents a unique opportunity to conduct population-based surveillance of this chronic condition and assess the impact of caring for children with CD on the health system.

**Objective:**

The objective of the study was to validate an algorithm based on health administrative data diagnostic codes to accurately identify children with biopsy-proven CD. We also evaluated trends over time in the use of health services related to CD by children in Ontario, Canada.

**Study design and setting:**

We conducted a retrospective cohort study and validation study of population-based health administrative data in Ontario, Canada. All cases of biopsy-proven CD diagnosed 2005–2011 in Ottawa were identified through chart review from a large pediatric health care center, and linked to the Ontario health administrative data to serve as positive reference standard. All other children living within Ottawa served as the negative reference standard. Case-identifying algorithms based on outpatient physician visits with associated ICD-9 code for CD plus endoscopy billing code were constructed and tested. Sensitivity, specificity, PPV and NPV were tested for each algorithm (with 95% CI). Poisson regression, adjusting for sex and age at diagnosis, was used to explore the trend in outpatient visits associated with a CD diagnostic code from 1995–2011.

**Results:**

The best algorithm to identify CD consisted of an endoscopy billing claim follow by 1 or more adult or pediatric gastroenterologist encounters after the endoscopic procedure. The sensitivity, specificity, PPV, and NPV for the algorithm were: 70.4% (95% CI 61.1–78.4%), >99.9% (95% CI >99.9->99.9%), 53.3% (95% CI 45.1–61.4%) and >99.9% (95% CI >99.9->99.9%) respectively. It identified 1289 suspected CD cases from Ontario-wide administrative data. There was a 9% annual increase in the use of this combination of CD-associated diagnostic codes in physician billing data (RR 1.09, 95% CI 1.07–1.10, P<0.001).

**Conclusions:**

With its current structure and variables Ontario health administrative data is not suitable in identifying incident pediatric CD cases. The tested algorithms suffer from poor sensitivity and/or poor PPV, which increase the risk of case misclassification that could lead to biased estimation of CD incidence rate. This study reinforced the importance of validating the codes used to identify cohorts or outcomes when conducting research using health administrative data.

## Introduction

Celiac disease (CD) is an autoimmune condition characterized by enteropathy resulting from exposure and immune response to gluten, a protein commonly found in wheat, rye, and barley.[1, 2] Once considered a rare disease, CD is now regarded as one of the most common autoimmune disorders, with an estimated prevalence of 1–3% in the overall population and 0.3 to 1.0% among the pediatric population.[3–6] Most studies of the epidemiology of CD were conducted in Europe, with incidence estimated between 2 and 54 cases per 100,000 patient-years (PY).[7–10]

Health administrative data in Ontario, Canada (the most populous province in Canada, population 13 million people, 38% of Canadian population) provides a unique opportunity to study the epidemiology of chronic diseases such as CD. Under Canada’s universal healthcare system, health administrative data were routinely generated for the health system utilization of all legal residents. With their large population coverage and ability to link between databases using a unique identification number, these data have been used extensively for epidemiology and health services research.[11–15] However, validation of health administrative data is key to ensure minimization of misclassification bias, proper interpretation of data and subsequent changes to health care delivery by potential users of such data. Algorithms to identify patients with chronic diseases should be validated against a reference data source, where the patients’ true disease status is known, in order to evaluate their performance in case ascertainment. [16]

The objective of the current study is to develop an algorithm to accurately identify children with biopsy-proven CD as biopsy is currently the standard of proof for diagnosing CD in North America.[1] The best algorithm was then used to estimate the use of health administrative data codes related to CD, and therefore the burden of pediatric CD in Ontario.

## Materials and methods

### Ethics and privacy statements

This study was approved by the Research Ethics Board of the Children’s Hospital of Eastern Ontario (CHEO), and the Privacy Officer at the Institute for Clinical Evaluative Sciences (ICES). The need for informed consent was waived by the ethics committee. All administrative data was provided to investigators using anonymous unique identifiers. No identifiable information can be published or shared beyond the approved investigators. The data set from this study is held securely in coded form at the Institute for Clinical Evaluative Sciences (ICES). While data sharing agreements prohibit ICES from making the data set publicly available, access may be granted to those who meet pre-specified criteria for confidential access, available at www.ices.on.ca/DAS. The full data set creation plan is available from the authors upon request.

### Data sources

We conducted a retrospective cohort and validation study linking clinical chart information to health administrative data. This study used population-based health administrative data from Ontario, Canada. Ontario is Canada’s largest and most populous province, comprising more than 12.8 million inhabitants. The Ontario Ministry of Health and Long-term Care (MOHLTC) provides universal health care coverage to all legal residents of Ontario (>99% of the population) through a single payer health care system. All Ontario physicians must submit claims for rendered services directly to MOHLTC for re-imbursement. These unique aspects of health care delivery in Ontario allow for comprehensive tracking of patient health care utilization and health outcomes by government agencies, and this data is made available for research purposes.

Six different databases, housed at ICES, were used in the current study. The full Ontario population data were available to study investigators and data analysts involved with this study. Individual patient data were linked deterministically using the ICES unique identification number, which is based on encrypted health card number. The Ontario Health Insurance Health Plan (OHIP) provided information on claims billed by primary care physicians and specialists in the Province of Ontario for clinical encounters and procedures. The Discharge Abstract Data (DAD) and the Same Day Surgery Database (SDS) from the Canadian Institute of Health Information (CIHI) were used to obtain information on hospitalization and same-day surgery. The ICES Physicians Database (IPDB) was used to ascertain information on the specialties of the physicians who billed the OHIP claims. The detailed coding scheme used to determine the specialties of the physicians were documented in S1 Table. The Registered Persons Database (RPDB) provided information on the demographics, residency and the OHIP eligibility by year for individuals who have ever received an OHIP card, while the 2006 Ontario Census Area Profile was used as the standard population to standardize the incidence rates tabulated in the study to the age and gender structure of the Canadian population.

### Validation cohorts

The true positive reference standard cohort was derived from review of patients’ charts at CHEO, the only pediatric health care center in Ottawa, Ontario, Canada. All pediatric endoscopies performed in the city of Ottawa and surrounding region are performed at CHEO, and therefore all biopsy-confirmed cases of childhood-onset CD were diagnosed there. The inclusion criteria for the true positive reference cohort included: CD patients aged 6 months to 14 years old, who were residing in the Census Metropolitan Area (CMA) of Ottawa between 2005 and 2011 with a valid health card number, and a complete medical chart available for review. The age range 6 months to 14 years old were chosen because aged 6 months represents the earliest possible age for expressing CD symptoms due to the introduction of solid food in diet; children over the age of 14 may start transitioning into adult care and stop seeking care from pediatric providers, thus by excluding them it decreased the risk of missing true positive cases in the reference cohort. Patients were excluded if they did not have continuous Ontario Health Insurance Plan (OHIP) eligibility from 2005 to 2011. This is to ensure that all of their medical services related to CD would be captured by the health administrative data, and minimize the potential for missing data resulting in errors during the algorithm derivation process. Potential CD cases from 2005 to 2011 were identified from CHEO using three different methods: (1) An electronic search of physician billing databases for the presence of International Classification of Disease, ninth version (ICD-9) code for CD (579.0); (2) electronic search of the pathology database for the presence of the Systematized Nomenclature of Medicine Clinical Terms (SNOMED CT) for duodenal biopsy associated with CD (“T64300 D6218”); (3) Patient lists from the CHEO dietician clinics for patients who received dietary counselling regarding a gluten-free diet. The medical charts of patients identified as potential cases by the 3 methods described above were reviewed by JC to confirm CD diagnosis. True biopsy-proven cases were defined as those whose histology findings received a Marsh classification of IIIa or above in the pathology report, following screening bloodwork suspicious of CD. [17] As standard practice, the Marsh classification was assigned by the pathologist interpreting the biopsy report in most cases. Where the classification was not available, the reviewer (JC) applied the Marsh criteria to the report. Ten percent of charts were reviewed in duplicate by a clinical expert (EIB) in blinded fashion and the weighted Kappa statistic was calculated to determine accuracy of diagnostic and Marsh classification. The following information was collected from patient charts using an *a priori* created data collection form: health card number (for linkage to health administrative data), date of birth, sex, diagnosis (CD or non-CD), date of diagnosis, and Marsh classification. Once all true positive biopsy-proven CD cases were confirmed with chart review, the health card number and data extracted from the health records were encrypted and transferred to ICES for deterministic linkage to the health administrative databases.

The true-negative reference cohort consisted of all patients determined not to have biopsy-proven CD based on the chart review, and all other children aged 6 months to 14 years living in the CMA of Ottawa between 2005 and 2011, identified from the Registered Person Database (RPDB). This strategy was used in previous algorithm derivation and validation studies in Ontario to determine a population-based true-negative reference standard and therefore calculate accurate predictive values. [12, 13]

### Algorithm derivation and testing

Various combinations of health services codes (outpatient physician visits, hospitalization, and procedure codes associated with the diagnosis of CD) were tested. The biopsy-proven CD case-identifying algorithm was planned to consist of two major components: (1) presence of a physician claim for an endoscopic procedure and (2) outpatient physician claims for visits associated with a diagnosis of CD. By varying the numbers and time intervals between different algorithm components, different algorithms were generated and tested.

Performance of the algorithms to accurately identify CD cases within health administrative data was tested against the true positive and negative reference cohorts. We calculated sensitivity, specificity, positive predictive value (PPV), and negative predictive value (NPV), with 95% confidence intervals (CI) using the efficient-score method corrected for continuity.[18] Accuracy of the algorithms was also tested in three different age groups (<12 years, <10 years, and <8 years) in order to account for differences in health service utilization in different ages.

### Using the algorithms to estimate the incidence of biopsy-proven CD in Ontario

We applied the selected algorithms with the best performance to the health administrative data to determine the incidence of biopsy-proven pediatric CD from 1995 to 2011. Age- and sex-standardized incidence per 100,000 children were calculated using each algorithm, with 95% confidence intervals (CI) determined using the Gamma method.[19] Annual Percentage Change (APC) of incidence was determined using Poisson regression, controlling age at diagnosis and sex. Potential interactions between year, age at diagnosis and sex were examined. Quadratic terms for year were added to the models to examine the linearity assumption between year and the incidence of CD.

## Results

### Participants

**Fig 1:**
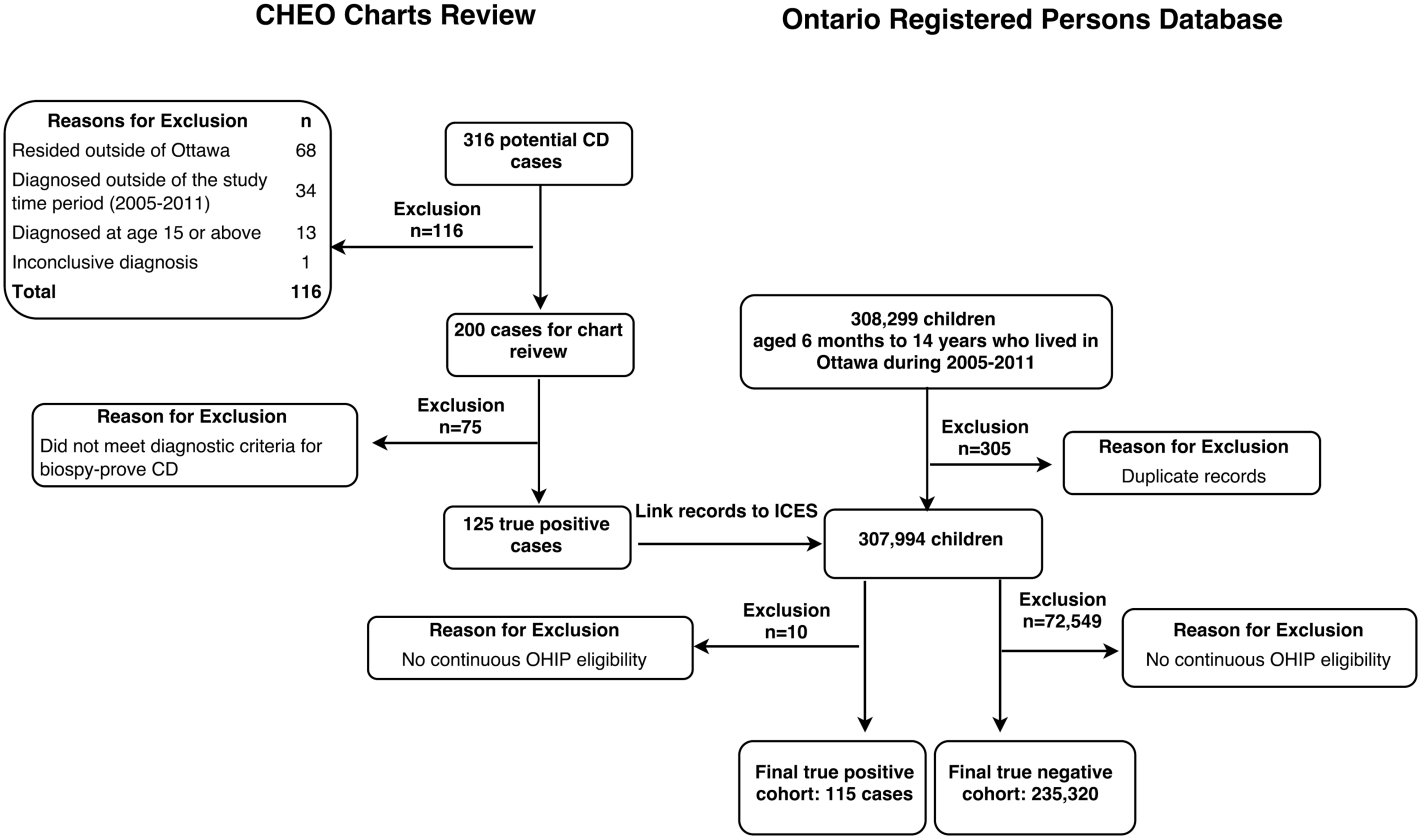
Study flowchart demonstrating the construction of true positive and true negative reference cohort.

Using the search strategy for true positive reference cases, 316 potential cases of pediatric CD cases between 2005 and 2011 were identified, of which 116 charts were excluded (Fig 1). The remaining 200 patients’ medical charts were fully reviewed and 125 cases of incident biopsy-proven CD were identified. In the 20 charts reviewed in duplicate, there was 100% agreement in the diagnosis of CD, and weighted kappa was 0.87 (95% CI: 0.75 to 0.99) for Marsh classification of the tissue biopsy.

Ten further patients were excluded after linkage to health administrative data as they did not have continuous OHIP eligibility during the study period. The final true positive reference cohort for algorithm derivation consisted of 115 patients. Of these, 62 cases were female (54%), and the median age of diagnosis was 7.4 years (IQR: 4.6 to 11.1 years). The majority of the CD patients’ duodenal biopsies were classified as Marsh 3b (70.4%), followed by Marsh 3a (15.7%) and Marsh 3c (13.9%).

Based on the RPDB, a total of 235,320 children lived in Ottawa during the study period and contributed to the true negative reference standard cohort.

### Algorithm derivation

Using the pre-determined algorithm structure, algorithms were derived and tested against the Ottawa reference cohort. The operating characteristics (sensitivity, specificity, PPV & NPV) of all of the tested algorithms were presented in the S2 Table. None of the algorithms achieved a high PPV while maintaining a satisfactory sensitivity. Examples of the tested algorithms and their operating characteristics are shown in Table 1. Two algorithms which differed in their source of endoscopy code were selected for further testing, as determined by face validity by clinical experts (DRM and EIB). The first algorithm, bolded in Table 1, termed the OHIP-based algorithm, included the presence of an OHIP endoscopy fee code for any indication plus ≥1 outpatient contacts with a gastroenterologist after the endoscopy (sensitivity 70.4% (95% CI 61.1 to 78.4%), specificity >99.9% (95% CI >99.9 to >99.9%), PPV 53.3% (95% CI 45.1 to 61.4), NPV >99.9% (95% CI >99.9 to >99.9%)). In this algorithm, the OHIP endoscopy billing claims are not specific to any indication. This is because in the OHIP billing database, only 1 diagnostic code is allowed to be included in the procedure claim. At the time of billing, the physician performing the procedure may not include the diagnostic code because he/she is not certain of the diagnosis yet. In fact, we observed that the sensitivities of the OHIP-based algorithms become extremely low when only OHIP-endoscopy claims with CD diagnostic codes were used.

**Table 1:** Operating characteristics of the algorithms with OHIP and SDS based endoscopy code.

Algorithm Number*	Algorithm Details	Sensitivity	Specificity	PPV	NPV
	**Algorithms with OHIP based endoscopy code**				
1—OHIP	Scope + 1 or more GI outpatient contacts	83.48 (75.14–89.51)	99.95 (99.94–99.96)	44.04(39.38–52.90)	99.99 (99.99->99.9)
2 –OHIP	Scope + 2 or more GI outpatient contacts in 1 year	46.96 (37.67–56.45)	99.98 (99.97–99.98)	50.00 (40.29–59.71)	99.97 (99.97–99.98)
3 –OHIP	Scope + 2 or more GI outpatient contacts in 2 years	51.30 (41.85–60.67)	99.98 (99.97–99.98)	51.75(42.25–61.1)	99.98(99.97–99.98)
4 –OHIP	Scope+ 2 or more GI outpatient contacts in 3 years	52.17 (42.70–61.50)	99.98 (99.97–99.98)	52.17 (43.70–61.50)	99.98 (99.97–99.98)
5 –OHIP	Scope + 2 or more GI outpatient contacts in 4 years	52.17 (42.70–61.50)	99.98 (99.97–99.98)	51.28 (41.91–60.56)	99.98 (99.97–99.98)
6 –OHIP	Scope + 2 or more GI outpatient contacts in 5 years	52.17 (42.70–61.50)	99.98 (99.97–99.98)	51.28 (41.91–60.56)	99.98 (99.97–99.98)
7 –OHIP	Scope + 1 or more GI outpatient contact or 1 hospitalization	86.96 (79.09–92.27)	99.94 (99.93–99.95)	41.67 (35.41–48.20)	99.99 (99.99->99.9)
8 –OHIP	Scope + 2 or more GI outpatient contacts or hospitalization in 1 year	50.43 (41.01–59.83)	99.98 (99.97–99.98)	50.88 (41.40–60.30)	99.98 (99.97–99.98)
9 –OHIP	Scope + 2 or more GI outpatient contacts or hospitalization in 2 years	54.78 (45.25–63.99)	99.98 (99.97–99.98)	52.50 (43.22–61.62)	99.98 (99.97–99.98)
10 –OHIP	Scope + 2 or more GI outpatient contacts or hospitalization in 3 years	57.39 (48.82–66.46)	99.98 (99.97–99.98)	53.66 (44.47–62.62)	99.98 (99.97–99.98)
11 –OHIP	Scope + 2 or more GI outpatient contacts or hospitalization in 4 years	57.39 (47.83–66.46)	99.97 (99.97–99.98)	52.80 (43.70–61.72)	99.98 (99.97–99.98)
12 –OHIP	Scope + 2 or more GI outpatient contacts or hospitalization in 5 years	57.39 (47.83–66.46)	99.97 (99.97–99.98)	52.80 (43.70–61.72)	99.98 (99.97–99.98)
**13 –OHIP**	**Scope + 1 GI or more outpatient contacts after scope**^1^	**70.43 (61.09–78.39)**	**99.97 (99.96–99.98)**	**53.29 (45.05–61.36)**	**99.99 (99.98–99.99)**
	**Algorithms with SDS based endoscopy code**				
**1—SDS**	**Scope + 1 or more GI outpatient contacts**^2^	**71.30 (62.00–79.16)**	**>99.9 (99.99->99.9)**	**92.13 (83.94–96.51)**	**99.99 (99.98–99.99)**
2 –SDS	Scope + 2 or more GI outpatient contacts in 1 year	38.26 (29.49–47.83)	>99.9 (>99.9->99.9)	93.62 (81.44–98.33)	99.97 (99.96–99.98)
3 –SDS	Scope + 2 or more GI outpatient contacts in 2 years	41.74 (32.73–51.31)	>99.9 (>99.9->99.9)	94.12 (82.77->98.47)	99.97 (99.96–99.98)
4 –SDS	Scope+ 2 or more GI outpatient contacts in 3 years	42.61 (33.54–52.17)	>99.9 (>99.9->99.9)	94.23 (83.08->98.50)	99.97 (99.96–99.98)
5 –SDS	Scope + 2 or more GI outpatient contacts in 4 years	42.61 (33.54–52.17)	>99.9 (>99.9->99.9)	94.23 (83.08->98.50)	99.97 (99.96–99.98)
6 –SDS	Scope + 2 or more GI outpatient contacts in 5 years	42.61 (33.54–52.17)	>99.9 (>99.9->99.9)	94.23 (83.08->98.50)	99.97 (99.96–99.98)
7 –SDS	Scope + 1 or more GI outpatient contact or 1 hospitalization	76.52 (67.53–83.71)	99.99 (99.98->99.9)	76.52 (67.53–83.71)	99.99 (99.98–99.99)
8 –SDS	Scope + 2 or more GI outpatient contacts or hospitalization in 1 year	41.74 (32.73–51.31)	>99.9 (>99.9->99.9)	92.31 (80.60–97.51)	99.97 (99.96–99.98)
9 –SDS	Scope + 2 or more GI outpatient contacts or hospitalization in 2 years	45.22 (36.01–54.75)	>99.9 (>99.9->99.9)	92.86 (81.87–97.69)	99.97 (99.97–99.98)
10 –SDS	Scope + 2 or more GI outpatient contacts or hospitalization in 3 years	47.83 (38.50–57.30)	>99.9 (>99.9->99.9)	93.22 (82.73–97.81)	99.97 (99.97–99.98)
11 –SDS	Scope + 2 or more GI outpatient contacts or hospitalization in 4 years	47.83 (38.50–57.30)	>99.9 (>99.9->99.9)	93.22 (82.73–97.81)	99.97 (99.97–99.98)
12 –SDS	Scope + 2 or more GI outpatient contacts or hospitalization in 5 years	47.83 (38.50–57.30)	>99.9 (>99.9->99.9)	93.22 (82.73–97.81)	99.97 (99.97–99.98)
13 –SDS	Scope + 1 or more GI outpatient contacts after scope	60.87 (51.30–69.70)	>99.9 (99.99->99.9)	92.11 (83.00–96.75)	99.98 (99.97–99.99)

*Algorithm numbers correspond with those in S1 Fig, which contains Venn diagrams demonstrating the overlap between cases identified using OHIP or SDS-based algorithms.

^1^Scope refers to the presence of an OHIP endoscopy fee code with any indication.

^2^Scope refers to the presence of an SDS endoscopy code indicated for CD.

The second algorithm, bolded in Table 1, termed the SDS-based algorithm, was: the presence of a SDS endoscopy code indicated for CD plus ≥1 outpatient contacts with a gastroenterologist (sensitivity 71.3% (95% CI 62.0 to 79.2%), specificity >99.9% (95% CI >99.9 to >99.9%), PPV 92.1% (95% CI 83.9 to 96.5%), NPV >99.9% (95% CI >99.9 to >99.9%)). Unlike the OHIP-based algorithm, the SDS-based algorithm’s endoscopy recorded is indicated for CD. This is because in SDS-endoscopy records, up to 20 diagnostic codes could be assigned to the record, and the record may be created at a later time when the result of the biopsy become available. The hospital coder may know the true diagnosis of the patient at the time of creating the record, thus more likely to apply to CD code to the SDS-endoscopy record. Venn diagrams for each of the 13 algorithms (S1 Fig) demonstrate the overlap between the OHIP-based and SDS-based algorithms in identifying cases.

To account for the potential health service utilization pattern differences among children in different age groups, we tested the algorithms based on the age at diagnosis with CD at: <12 years, <10 years, and <8 years. The accuracies of the algorithms did not differ substantially in these age groups (S2 Table).

### Use of algorithms to calculate incidence of CD

**Fig 2:**
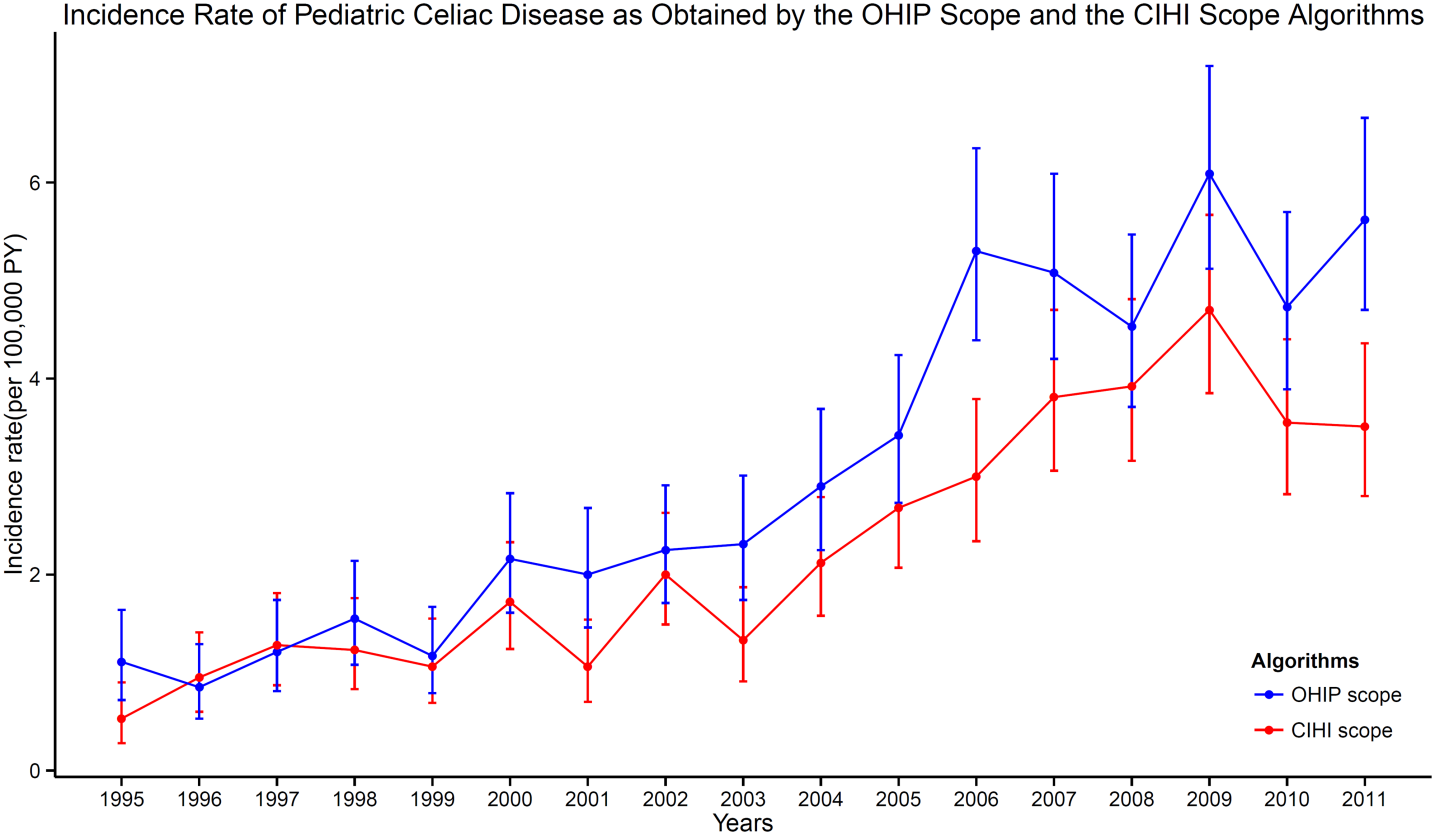
Incidence rate of pediatric celiac disease as obtained by the OHIP-based ad SDS-based algorithms.

The two selected algorithms were applied to the Ontario health administrative data of all children aged 6 months to 14 years who resided in Ontario during 1995 to 2011 (n = 5,736,813). The OHIP-based algorithm ascertained 1,289 cases while the SDS-based algorithm ascertained 948 cases. The annual incidence estimate, standardized to the 2006 Canadian census population based on age and sex structure, is presented in Fig 2.

Based on the OHIP-based algorithm, we observed an annual percentage change of +9% (IRR: 1.09, 95% CI 1.07 to 1.10; P<0.001) from 1995 to 2011. An interaction between age at diagnosis and the year of diagnosis was detected from the model based on the SDS-based algorithm. The annual increase in CD incidence from 1995 to 2011 was still observed, but the rate of increase varied based on the age of diagnosis. The estimates for both models were summarized in Table 2.

**Table 2:** Trends in incidence over time by poisson regression, adjusting for gender and age of diagnosis.

Variables	Estimates (95% CI)	Rate Ratio (95% CI)^1^	P-value
**OHIP-based Algorithm**			
Intercept	1.07 (0.90 to 1.25)	2.91 (2.45 to 3.50)	<0.001
Year of diagnosis	0.08 (0.07 to 0.10)	1.09 (1.07 to 1.10)	<0.001
Gender (Male)	-0.33 (-0.45 to -00.22)	0.72 (0.64 to 0.80)	<0.001
Age of diagnosis	-0.06 (-0.07 to -0.04)	0.94 (0.93 to 0.96)	<0.001
**SDS-based Algorithm**			
Intercept	1.23 (0.95 to 1.50)	3.41 (2.60 to 4.48)	<0.001
Year of diagnosis	0.04 (0.02 to 0.07)	1.04 (1.02 to 1.07)	<0.001
Gender (Male)	-0.36 (-0.48 to -0.24)	0.70 (0.62 to 0.79)	<0.001
Age of diagnosis	-0.09 (-0.12 to -0.05)	0.91 (0.88 to 0.95)	<0.001
Year of diagnosis x Age of diagnosis	0.004 (0.0004 to 0.007)	1.004 (1.0004 to 1.007)	0.028

^1^Calculated by exponentiating the estimates

## Discussion

The current study evaluated the feasibility of using health administrative data to capture children with biopsy-proven CD, and explored the trends in the use of CD diagnostic codes in these data over the past two decades in Ontario. Our findings demonstrated that Ontario health administrative data is suboptimal for accurate classification of biopsy-proven CD among children, at least with currently available data structure comprising mostly outpatient physician bill coding and hospitalization data. All of the algorithms derived in the study suffered from low sensitivity, and/or low PPV. We applied two of the test algorithms to the Ontario health administrative data to illustrate the effect of using algorithms for incidence rate estimation. The OHIP-based and SDS-based algorithms, differing by the source of their endoscopy procedural code, both detected an increased trend in health services use associated with CD diagnostic code during the study time period. This likely reflects increased health services utilization for CD and gluten-related disorders. However, due to the suboptimal accuracy of the two algorithms, we cannot make firm conclusions regarding the trends in pediatric CD incidence over the past two decades in Canada.

To ensure maximum accurate identification of patients, we attempted to validate algorithms against a reference standard cohort consisting of the entire Ottawa pediatric population. We used this cohort to test the performance of different identification algorithms prior to applying them to Ontario health administrative data. Two previous studies evaluated the accuracy of the health administrative data codes for identification of patients with CD. One study from the UK only used a positive reference cohort of 38 patients without a negative reference standard [20], while a Danish study used patients from a single hospital as reference.[5] Both reference cohorts were not accurate representations of the overall population, and as a result, performance of the codes in the overall population cannot be assumed. Specifically, since the studies lacked a population-based true negative reference, the prevalence of CD in their reference cohort would be higher than that found in the general population. This could result in the algorithms demonstrating inflated PPV.[16] In our study, the defined Ottawa-based true positive and negative populations created a reference standard cohort with the prevalence of disease reflective of the true prevalence in the population, thus producing more accurate estimates for the operating characteristics of the algorithms.

To ensure that the incident CD cases were biopsy proven, we constructed our algorithms based on a pre-determined two-step structure proposed by expert clinicians. The structure of the algorithms resembled the disease investigation process of children suspected of having CD. The algorithms require the presence of an endoscopy procedural code followed by various numbers of outpatient contacts or hospital discharges for CD. We observed dramatic differences in algorithms performance based on the source of endoscopy procedural code. Specifically, algorithms with a SDS-based endoscopy code achieved a significantly higher PPV when compared to the algorithms based on OHIP-based endoscopy code. This may be secondary to differences in coding practices between the two health administrative databases. The OHIP endoscopy billing claims were typically completed by the physicians (or representatives) after the procedure, but prior to interpretation of biopsies being available. In contrast, the SDS endoscopy records were completed by professional hospital coders up to one month after the procedure. These coders may have had access to biopsy results at the time of data entry. As a result, the SDS-based algorithm may be more accurate in distinguishing CD and non-CD patients, resulting in elevated PPV. While most pediatric endoscopies performed at large pediatric hospitals will be recorded in the SDS database, those conducted in community practices or private endoscopy clinics may not be recorded in SDS. While these likely represent a minority of procedures in Ontario children, such differences in coding practices could jeopardize the generalizability of the SDS-based algorithm. Therefore, despite the adequate performance of the SDS-based algorithm, we are skeptical that it could be applied to the full province without misclassification bias resulting in underestimation of incidence estimates. On the other hand, the algorithm which used OHIP-based endoscopy fee codes performed poorly, likely resulting in overestimation of incidence estimates. It is possible that the true number of incidence cases lies somewhere between the estimates produced by the two algorithms.

In Ontario, any physician may follow a patient with CD to monitor overall health, including gastroenterologists, nurse practitioners, pediatricians, or family physicians. These providers are registered with OHIP and therefore health care contacts with any of these providers would be contained within the administrative databases. In the current study, the second component of the algorithms evaluated specified the number of outpatient CD-related visits to physicians. Following the initial diagnosis period, children with biopsy-proven CD in Ottawa did not frequently have more than one outpatient physician visit associated with a CD diagnostic code. Of all patients diagnosed with CD (true positive reference cohort), fewer than 70% had two or more outpatient visits to a physician or hospitalizations associated with CD diagnostic code. When only clinical encounters from gastroenterologists were considered, fewer than 60% had two or more CD visits. This indicated that CD did not result in cause-specific health service utilization once the diagnosis was established and therapeutic diet initiated. This is supported by a recent study which reported that pediatric CD patients had significantly lower health services use after diagnosis.[21] In addition, it is possible that patients would only be followed by registered dietitians after diagnosis (patient choice). These visits would not be contained within health administrative data, since dietitians cannot bill their service through OHIP.

While the algorithms performed sub-optimally, they provide useful information regarding the use of health services claims codes for CD in Ontario. Therefore, we can infer that since the number of cases identified by both algorithms rose between 1995 and 2011, use of the health care system related to biopsy proven CD has increased substantially over the past two decades. This increase in apparent number of CD “cases” may have been caused by increased patient or physician awareness of the condition, better disease detection and greater numbers of pediatric gastroenterologists following current recommendations of performing endoscopies to confirm CD diagnosis. However, some family chose to forego endoscopic evaluation for diagnosis confirmation after receiving positive results from screening blood tests. This has the potential of underestimating the true incidence or CD as these unconfirmed cases may not be included in the estimates. A pediatric longitudinal study from UK reported that close to 90% of CD cases were left undiagnosed in the population.[22] However, the increased coding for CD may also be related to increased awareness of non-celiac gluten sensitivity (NCGS) driven by the popular press and reported in new scientific literature. [23] Patients may have described gluten sensitivity to their physician at health care visits, resulting in an outpatient physician billing claim with associated CD diagnosis, since no ICD code exists for NCGS. While we attempted to avoid this misclassification by including endoscopy codes to identify only biopsy-proven CD, those with negative biopsies may still have been labelled as having CD. Better education of physicians on the difference between CD and NCGS may avoid these coding practices.

This study is subject to a number of limitations. The algorithms were derived and tested in Ottawa, but not in other regions in the province of Ontario. Therefore, the algorithms may perform differently in other regions where differing practice patterns exist. However in Ontario, the majority of pediatric gastroenterology care and endoscopies of children is centralized in six large pediatric academic health centers, with very few community-based pediatric gastroenterology practices existing.[24] In addition, pediatric gastroenterologists in Ontario academic centers are paid via alternate funding plans (AFPs) and are not reimbursed based on fee-for-service billing. Therefore, the patterns of care of Ottawa patients should be relatively similar to those in other centers. It is important to emphasize that the validated algorithms in the study were generated from health administrative data, therefore, they may not be applicable in other jurisdiction/countries that use other data sources for chronic disease surveillance (e.g. primary care data). Another significant weakness of this study is that both pathology and serological laboratory results are not available in Ontario health administrative data. It is possible that if the results of such tests as the anti-tissue transglutaminase antibody serology were available, we could have validated a more accurate algorithm. In future, linkage of these test results and other clinical data from electronic health records or other data sources could supplement health administrative data and result in more accurate identification of patients.

## Conclusions

In summary, the algorithms derived to identify children with biopsy-proven CD from within Ontario health administrative data demonstrated suboptimal performance. However, there was a clear and significant increase in the use of CD diagnostic codes by Ontario physicians. This study demonstrated the limitations of using health administrative data to derive a cohort of children with CD. Not all diseases can be accurately identified using health administrative data codes. Our study emphasizes the importance of evaluating the accuracy and completeness of codes/algorithms used to identify patients from within health administrative data in order to reduce error potentially resulting in misclassification bias.

## Supporting information

S1 Table Supplementary Table 1. Definition of specialty, and code list for endoscopy used in this study.
(DOCX)

S2 Table Supplementary Table 2. Diagnostic characteristics of various algorithms (with 95% confidence intervals) to identify children with biopsy-proven celiac disease based on validation study.
(XLS)

S1 Fig Supplementary Figure 1. Venn diagram noting overlap between cases identified using all OHIP and SDS based algorithms noted in Table 1. Note: Due to privacy regulations, all cell sizes with five or fewer subjects are indicated as ‘<6’ and those outside the union of identified cases were rounded to the nearest 10.
(TIF)
