# Supplementary material for: Validation of an algorithm to identify children with biopsy-proven celiac disease from within health administrative data: An assessment of health services utilization patterns in Ontario, Canada
Source: PLoS One. 2017 Jun 29;12(6):e0180338. doi: 10.1371/journal.pone.0180338 (PMC5491178; doi:10.1371/journal.pone.0180338)
Supplement: S1 Table — Definition of specialty, and code list for endoscopy used in this study. (DOCX) [file pone.0180338.s001.docx]

Supplementary Table 1. Definition of specialty, and code list for endoscopy used in this study.

| **SPECIALTY DEFINITION: GASTROENTEROLOGIST** | |
| --- | --- |
| Classification | Definition |
| Adult Gastroenterologist | Considered an adult gastroenterologist specialist if: any internal medicine specialist with certification in gastroenterology or any internal medicine specialist who has records for >100 OHIP procedural codes for endoscopy in one year. Procedural codes listed below |
| Pediatric Gastroenterologist | Considered a pediatric gastroenterologist specialist if: any pediatrician with certification in gastroenterology or any pediatrician who has records for >5 OHIP procedural codes for endoscopy in one year. Procedural codes listed below. |
| **SPECIALTY DEFINITION: GENERALIST** | |
| **Classification** | **Definition** |
| Family Physician or General Practitioner | Considered a family physician if: certified in IPDB as a family physician. |
| General Pediatrician | Considered a general pediatrician if: certified in IPDB as pediatrics, but not Pediatric Gastroenterologist |
| General Internist | Considered a general internist if: certified in IPDB as internal medicine, but not as Adult Gastroenterologist |
| Surgeon | Considered a surgeon if: certified in IPDB as a general surgeon. |
| **PROCEDURAL CODES FOR COLONOSCOPY OR SIGMOIDOSCOPY (FROM OHIP DATABASE) (used only to distinguish gastroenterologist from non-gastroenterologist specialty)** | |
| Z496 | Presence of signs or symptoms – sigmoid to descending colon |
| Z497 | Confirmatory colonoscopy – sigmoid to descending colon |
| Z498 | Surveillance colonoscopy – sigmoid to descending colon |
| Z499 | Colonoscopy – absence of signs or symptoms, family history of colon cancer in a first degree relative sigmoid to descending colon |
| Z535 | Intestines – endoscopy-sigmoidoscopy with or without anoscopy |
| Z536 | Intestines – endoscopy-sigmoidoscopy with biopsy |
| Z555 | Intestines – endoscopy-colonoscopy into descending colon |
| **PROCEDURAL CODES FOR UPPER ENDOSCOPY (FROM OHIP DATABASE)** | |
| Z399 | Endoscopy (elective) |
| Z400 | Endoscopy (for active bleeding) |
| Z527 | Gastroscopy (may include biopsies, photograph and removal of polyps) |
| Z547 | Gastroscopy (with removal of foreign body) |
| Z558 | ERCP with cannulation of common bile duct and/or pancreatic duct |
| Z560 | Duodenoscopy |
| Z561 | ERCP with cannulation of common bile duct and/or pancreatic duct |
| Z749 | Endoscopy (subsequent procedure, within 3 months following previous endoscopic procedure) |
| Z584 | Small bowel push enteroscopy |
| **PROCEDURAL CODES FOR COLONOSCOPY OR SIGMOIDOSCOPY (FROM CIHI SDS DATABASE)** | |
| 0115 | Endoscopy of small intestine through existing artificial stoma |
| 0116 | Other nonoperative endoscopy of small intestine |
| 5791 | Brush biopsy of small intestine |
| 5792 | Other biopsy of small intestine |
| 5795 | Biopsy of intestine, unqualified |

| CIHI SDS: Canadian Institute for Health Information Same Day Surgery; ERCP: endoscopic retrograde cholangiopancreatography; IPDB: ICES Physician Database; OHIP: Ontario Health Insurance Plan. |
| --- |
