# Supplementary figures and images for: Validation of an algorithm to identify children with biopsy-proven celiac disease from within health administrative data: An assessment of health services utilization patterns in Ontario, Canada
Source: PLoS One. 2017 Jun 29;12(6):e0180338. doi: 10.1371/journal.pone.0180338 (PMC5491178; doi:10.1371/journal.pone.0180338)

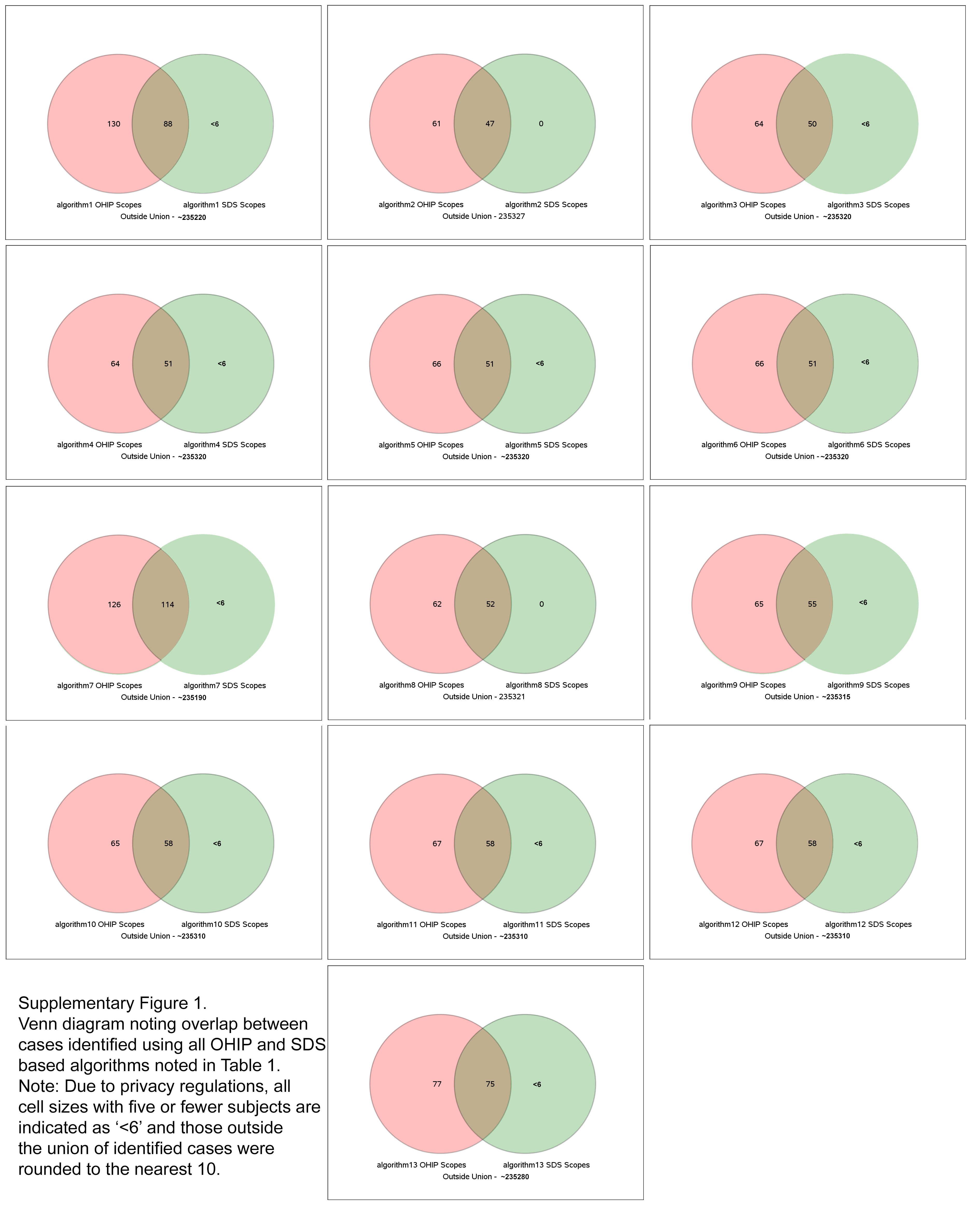

Supplement: S1 Fig — Venn diagram noting overlap between cases identified using all OHIP and SDS based algorithms noted in Table 1. Note: Due to privacy regulations, all cell sizes with five or fewer subjects are indicated as ‘<6’ and those outside the union of identified cases were rounded to the nearest 10. (TIF) [file pone.0180338.s003.tif]
